# Cortical grey matter volume and sensorimotor gating in schizophrenia

**DOI:** 10.1016/j.cortex.2007.11.007

**Published:** 2008-10

**Authors:** Veena Kumari, Dominic Fannon, Mark A. Geyer, Preethi Premkumar, Elena Antonova, Andrew Simmons, Elizabeth Kuipers

**Affiliations:** a Department of Psychology, Institute of Psychiatry, King's College London, London, UK; b Division of Psychological Medicine, Institute of Psychiatry, King's College London, London, UK; c Department of Psychiatry, University of California at San Diego, La Jolla, CA, USA; d Centre for Neuroimaging Sciences, Institute of Psychiatry, King's College London, London, UK; e NIHR Biomedical Research Centre for Mental Health, South London and Maudsley NHS Trust, London, UK

**Keywords:** Prepulse inhibition, Startle, Schizophrenia, Voxel-based morphometry, Frontal cortex

## Abstract

Prepulse inhibition (PPI) of the startle response, a cross-species measure of sensorimotor gating, provides a valuable tool to study the known inability of a large proportion of individuals with schizophrenia to effectively screen out irrelevant sensory input. The cortico-striato-pallido-thalamic circuitry is thought to be responsible for modulation of PPI in experimental animals. The involvement of this circuitry in human PPI is supported by observations of deficient PPI in a number of neuropsychiatric disorders that are characterised by abnormalities at some level in this circuitry, and findings of recent functional neuroimaging studies in healthy participants. The current study sought to investigate the structural neural correlates of PPI in a sample of 42 stable male outpatients with schizophrenia. Participants underwent magnetic resonance imaging (MRI) at 1.5 T and were assessed (off-line) on acoustic PPI using electromyographic recordings of the orbicularis oculi muscle beneath the right eye. Optimised volumetric voxel-based morphometry implemented in SPM2 was used to investigate the relationship of PPI (prepulse onset-to-pulse onset interval 120 msec) to regional grey matter (GM) volumes. Significant positive correlations were obtained between PPI and GM volume in the dorsolateral prefrontal, middle frontal and the orbital/medial prefrontal cortices. Our findings are consistent with (a) previous suggestions of susceptibility of PPI to cognitive processes controlled in a ‘top down’ manner by the cortex and (b) the hypothesis that compromised neural resources in the frontal cortex contribute to reduced PPI in schizophrenia.

## 1 Introduction

Prepulse inhibition (PPI) of the startle response refers to a reliable reduction in the amplitude of the startle response to a strong sensory stimulus, the pulse, if this is preceded shortly (30–500 msec) by a weak non-startling stimulus, the prepulse (Graham, 1975). PPI is believed to be an operational measure of a sensorimotor filtering system (Geyer et al., 1990). Since the first demonstration by Braff et al. (1978), a large number of studies have confirmed that individuals with schizophrenia, on average, show reduced PPI compared to healthy individuals (Braff et al., 2001, 2005; Meincke et al., 2004; Swerdlow et al., 2006; Kumari et al., 2007). The cognitive overload resulting from reduced sensorimotor gating is thought to give rise to some of the complex clinical symptoms associated with this disorder (Geyer et al., 1990; Braff, 1993).

Animal studies demonstrate that PPI is modulated by the cortico-striatal-pallido-thalamic (CSPT) circuitry involving the prefrontal cortex, thalamus, hippocampus, amygdala, nucleus accumbens, striatum, ventral pallidum, globus pallidus, and subpallidal efferents to the pedunculopontine nucleus (reviews, Swerdlow and Geyer, 1998; Swerdlow et al., 2001). Pharmacological and surgical challenges to substrates of the CPST circuitry reliably produce changes in PPI in experimental animals (reviews, Geyer et al., 2001; Swerdlow et al., 2001).

Previous studies have utilised structural magnetic resonance imaging (MRI) volumetry to study the neural correlates of higher order cognitive functions in healthy (e.g. Maguire et al., 2000; Ettinger et al., 2002, 2005; Sanfilipo et al., 2002) and clinical populations, including schizophrenia (review, Antonova et al., 2004; Ettinger et al., 2004). Recent data, for example, demonstrating a relationship between the behavioural measures of specific frontal lobe function and regional frontal lobe volumes (Sanfilipo et al., 2002; Ettinger et al., 2005) and between memory and hippocampal volumes (Maguire et al., 2000), clearly demonstrate that structural properties of the specific brain regions are closely associated with behavioural measures of their function. Extending this approach to study the brain basis of human PPI, our previous study demonstrated a significant relationship between PPI and grey matter (GM) volume on a highly spatially localised scale in the hippocampal, striatal, thalamic, temporal and frontal regions in 24 healthy participants (Kumari et al., 2005). Although no study has as yet examined the contribution of structural brain volume to PPI in patients with schizophrenia, a degree of volumetric change has been demonstrated in those brain regions relevant to PPI (review, Shenton et al., 2001).

The present study aimed to investigate the structural brain correlates of acoustic PPI in a clinically stable group of men with schizophrenia. As in our previous investigation of healthy participants (Kumari et al., 2005) we employed voxel-based morphometry (VBM) which allows the examination of correlations between GM volume and behavioural measures on a voxel-by-voxel basis across the entire brain rather than limiting the search to certain regions of interests (ROIs) (Gaser and Schlaug, 2003). In light of our previous observations in healthy people (Kumari et al., 2005) we hypothesized that less GM volume in the frontal, hippocampus/temporal lobe, striatum, thalamus, and possibly parietal regions would be associated with lower PPI in patients with schizophrenia.

## 2 Materials and methods

### 2.1 Participants

This study included 50 men with a diagnosis of schizophrenia or schizoaffective disorder, from 42 of whom usable psychophysiology and imaging data were acquired for the current investigation. Of those not included, four patients were startle non-responders (mean amplitude < 10 μV), two patients could not be tested on PPI for practical reasons, and imaging data were unusable for two patients. All patients were (i) recruited from the South London and Maudsley NHS Foundation Trust, (ii) on stable doses of antipsychotic medication for at least three months prior to taking part, (iii) in a stable (chronic) phase of the illness, and (iv) were living in the community or long stay/rehabilitation wards. These participants had not taken part in any of our previous PPI studies. Table 1 shows demographic and clinical characteristics of the study group. The clinical, psychophysiological (PPI of the startle response) and imaging data were acquired within a period of 2–7 days for each individual.

The study procedures were approved by the joint research ethics committee of the Institute of Psychiatry and the South London and Maudsley NHS Foundation Trust. All participants provided written informed consent and were reimbursed for their time and travel.

### 2.2 Diagnoses and clinical assessments

Clinical diagnoses were made by an experienced consultant psychiatrist (DF) blind to the subsequent MRI and PPI measures, using the Structured Clinical Interview for DSM-IV (SCID; First et al., 1995). Symptoms were rated by the same psychiatrist using the Positive and Negative Syndrome Scale (PANSS) (Kay et al., 1987). In addition, predicted IQ was measured using the National Adult Reading Test (NART) (Nelson and Willison, 1991) and current IQ was measured using the Wechsler Abbreviated Scale of Intelligence (two-test version; Wechsler, 1999) for sample characterization purposes.

### 2.3 PPI: paradigm, startle response measurement and scoring

A commercially available human startle response monitoring system (Mark II, SR-Lab, San Diego Instruments, San Diego, California) was used to generate and deliver the acoustic stimuli, and to record and score the electromyographic (EMG) activity for 250-msec starting from the onset of the acoustic startle stimulus. Acoustic stimuli were presented to participants binaurally through headphones. The pulse-alone stimulus was a 40-msec presentation of 114-dB (A) white noise and the prepulse stimulus a 20-msec presentation of 85-dB (A) white noise, both over 70-dB (A) continuous background noise. The session began with a 5 min acclimatization period consisting of 70-dB (A) continuous white noise. Participants received four blocks of 12 trials each, after an initial pulse-alone trial. Each block consisted of three pulse-alone trials, three prepulse trials with a 30-msec prepulse-to-pulse (onset-to-onset) interval, three prepulse trials with a 60-msec prepulse-to-pulse interval, and three prepulse trials with a 120-msec prepulse-to-pulse interval presented to individuals in a pseudorandom order with a mean inter-trial interval of 15 sec (range 9–23 sec).

The experimental procedures for recording and scoring the startle reflexes were identical to those reported previously (e.g. Kumari et al., 2005). The eyeblink component of the startle was indexed by recording EMG activity of the orbicularis oculi muscle directly beneath the right eye, by positioning two miniature silver/silver chloride electrodes. Recorded EMG activity was band-pass filtered, as recommended by the manufacturer and a 50-Hz filter used to eliminate 50-Hz interference. The EMG data were at first inspected on a trial-to-trial basis (to exclude erroneous trials for a particular person) and then scored by the system's analytic programme for response amplitude (in arbitrary units; 1 unit = 2.62 μV) and latencies (in msec). Responses (<4%) were rejected if the onset and peak latencies differed by more than 95 msec or when the baseline values shifted by more than 50 units.

Participants were told that the experiment was to measure their reaction to a number of noise-bursts, but no specific instructions were given to attend or ignore them. They were requested to keep their eyes open during the experiment. There was no explicit restriction on smoking intake prior to testing but care was taken not to take participants to the startle laboratory for about 25 min after they had a cigarette, in order to prevent a state of smoking withdrawal or a heavy intake during the testing session that may transiently affect PPI (Kumari et al., 2001).

PPI was computed for each participant separately for each trial type as (*a* − *b*/*a*) × 100, where “*a*” is the pulse-alone amplitude and “*b*” is the amplitude over prepulse trials. Percent of PPI was used rather than the absolute value of PPI (i.e. arithmetic difference between pulse-alone and prepulse trials), since this eliminates the influence of individual differences in startle responsiveness.

As in our previous study of healthy participants (Kumari et al., 2005), PPI at the 120-msec prepulse-to-pulse interval was chosen as the main dependent measure for hypothesis testing because (a) this interval produces the maximum PPI in humans (i.e. allows the maximum power in terms of range of scores) and (b) is the most frequently used interval in clinical studies (reviews, Braff et al., 2001; Kumari and Ettinger, 2005). We chose to study 120-msec PPI rather than the mean PPI across all prepulse-to-pulse intervals because the neural correlates of PPI may differ somewhat at different intervals, and focussing on a particular interval would facilitate a precise comparison of the results of future studies on this topic.

### 2.4 MRI

#### 2.4.1 Data acquisition

Structural MRI brain scans were acquired using the 1.5 T GE NV/i Signa system (General Electric, Milwaukee WI, USA) at the Maudsley Hospital, London. A quadrature birdcage head coil was used for RF transmission and reception. Head movement was limited by foam padding within the head coil and a restraining band across the forehead. Initially, a series of sagittal and axial fast gradient echo scout images were acquired in order to correct for head tilt and to orient subsequent images relative to the anterior-commissure/posterior-commissure line and the interhemispheric fissure (TR = 200 msec, TE = 4.2 msec, *θ* = 90°, field of view = 24 cm, slice thickness = 5 mm, slice gap = 2.5 mm, 256 × 192 acquisition matrix, one data average). The whole brain was then scanned with a 3-D inversion recovery prepared fast spoiled GRASS T1-weighted dataset. These T1-weighted images were obtained in the coronal plane with 1.5-mm contiguous sections. TR was 18 msec, TI was 450 msec, TE was 5.1 msec and the flip angle was 20° with one data average and a 256 × 256 × 128 voxel matrix.

#### 2.4.2 MRI pre-processing

Structural images were converted into ANALYZE format (ANALYZE software, BRU, Mayo Foundation, Rochester, MN). A manual determination of the AC–PC line was performed for all images prior to pre-processing. The images were pre-processed as required for the optimised protocol for VBM devised and validated by Good et al. (2001) within the statistical parametric mapping software package (SPM2, Wellcome Department of Cognitive Neurology, University College London, London, UK; http://www.fil.ion.ucl.ac.uk/spm), running in Matlab 6.1 (MathWorks, Natick, MA). An overview of the procedures is as follows.

#### 2.4.3 Customised template creation

Customised templates of the whole brain, GM, white matter (WM) and cerebro-spinal fluid (CSF) were created. For the creation of the customised whole brain (T1) template, the images were spatially normalized to the standard SPM2 T1 template using a 12-parameter affine transformation. These normalized images were then smoothed with an 8-mm full width at half maximum (FWHM) isotropic Gaussian kernel, and averaged to create a customised T1 template. The normalized T1 images were then segmented into their GM, WM and CSF components using the GM, WM and CSF probability maps inherent to SPM2. The resultant tissue segments were automatically cleaned to remove non-brain tissue and smoothed with an 8-mm FWHM isotropic Gaussian kernel, normalized using affine transformation with sinc interpolation algorithm and averaged to derive GM, WM and CSF probability maps. To reduce the partial volume problem and ensure optimal tissue segmentation, all images for the templates were written out with 1 × 1 × 1 mm voxel size.

#### 2.4.4 Deriving and applying optimised normalization parameters

The structural scans were processed using the customised whole brain and tissue probability templates. The first step entailed a segmentation of the original images in native space, registering to the customised tissue probability map and correcting for image inhomogeneity, followed by the automatic brain extraction and cleaning procedure to remove non-brain tissue. The second step involved spatial normalization of the original images to the customised whole brain template using 12-parameter linear and 7 × 8 × 7 discrete cosine transform basis function non-linear transformation (Ashburner and Friston, 1999), with parameters determined from the images derived from the first step, and resliced to 1 × 1 × 1 mm voxel size to yield more accurate subsequent tissue segmentation. The spatially normalized images were then segmented into the three tissue compartments using the customised GM, WM and CSF templates. Brain extraction and cleaning procedures were re-applied to the segmented normalized GM images to further remove extraneous brain tissue. Since the volume of some brain regions may shrink or expand as a result of non-linear spatial normalization, the cleaned GM images were modulated, i.e., the voxel values of each segment were multiplied by the Jacobian determinants of the deformation matrix derived during the spatial normalization step to ‘restore’ the original volume of each GM segment. Lastly, the GM and WM segments were smoothed using a 12-mm FWHM isotropic Gaussian kernel to make the data conform to the Gaussian field model, underlying the structural inferences as applied in SPM2. This serves to render the data more normally distributed and to reduce the effects of individual variation in sulcal/gyral anatomy (Ashburner and Friston, 2000).

### 2.5 Data analysis

The relationship of PPI to current age, IQ, age at illness onset, and symptoms was examined using Pearson's correlations. Simple linear regression of 120-msec PPI to GM volume maps was then performed at each voxel within SPM2. The resulting statistical parametric maps were thresholded at *t* = 3.31, *p* < .001, uncorrected only allowing clusters extending over 25 contiguous significant voxels (each with threshold *t* = 3.31), as these are unlikely to occur by chance (Sowell et al., 1999). If these clusters were in a region which was hypothesized to be related to PPI, small volume correction (SVC) was applied to determine whether a cluster is significant after correcting for multiple comparisons within a locally defined volume rather than the whole brain. A 15 mm radius sphere centred on the maxima voxel was used in SVC analyses. These statistical procedures and thresholds are the same as used previously to identify PPI–GM volume relationships in a healthy group (Kumari et al., 2005).

Next, the values representing the percentage of total grey volume under a smoothing kernel relative to the total GM volume for each participant at the maxima voxel of the (three) regions that showed an association with PPI (see Section 3) were extracted. These values were examined (a) using multiple regression to assess the total variance in PPI explained by them and (b) using Pearson's correlations for any associations with age, age at illness onset and current symptoms in order to exclude confounding (SPSS, v15). We further examined the correlations between the percentage of GM volume in these regions and (a) 120-msec PPI after controlling for symptoms (total PANSS score) and mean startle amplitude using partial correlations and (b) with 30-msec and 60-msec PPI using Pearson's correlations. Finally, we evaluated the strength of correlations between the percentage of GM volume in the regions that showed an association with PPI across the entire sample after excluding the patients under drugs other than atypical antipsychotics alone (*n* = 36) using Pearson's correlations.

The total GM volume for each patient was calculated (in millilitre) from the unsmoothed modulated segmented images (which have the same GM volume as the segmented images in native space) and examined for its association with PPI across the entire sample.

## 3 Results

As expected, and replicating numerous previous reports (review, Braff et al., 2001), the 120-msec prepulse-to-pulse interval produced the maximum PPI (Table 1) which was strongly positively correlated with PPI at 30-msec (*r* = .693, *p* < .001) and 60-msec intervals (*r* = .640, *p* < .001). PPI did not correlate with current age, IQ, age at illness onset, positive symptoms, negative symptoms or general psychopathology scores in this sample (all *p* > .05 uncorrected for multiple comparisons).

### 3.1 Correlations with GM volume

**Fig. 1:**
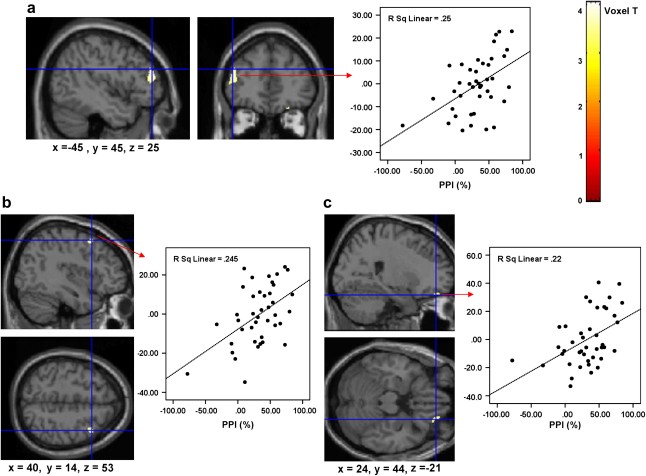
Group correlation map thresholded at *p* < .001 in SPM2 showing positive associations between PPI and GM volumes in the left prefrontal cortex (1a), right middle frontal cortex (1b), and the orbital/medial prefrontal cortex (1c) imposed on the SPM2 single brain. Associated scatter plots (1a–c) show the relationships between the percentage GM volume at the maxima voxel (*Y*-axis) in the localised region and PPI (*X*-axis) for each participant. Left hemisphere is shown on the left. Colours represent voxel level *t*-values and strength of the correlation with PPI.

PPI was positively correlated with GM volume in the left dorsolateral prefrontal cortex (centred at *x* = −45, *y* = 45; *z* = 25; SPM2 derived *T* = 4.05; *p* < .001 uncorrected, .015 corrected; see Fig. 1a), the right middle frontal cortex (centred at *x* = 40, *y* = 14; *z* = 53; *t* = 3.61; *p* < .001 uncorrected, .035 corrected; Fig. 1b) and the right orbital/medial prefrontal cortex (centred at *x* = 24, *y* = 44; *z* = −21; *t* = 3.55; *p* = .001 uncorrected; .04 corrected; Fig. 1c). As demonstrated in Fig. 1 (scatter plots) one patient showed marked facilitation, rather than inhibition of the startle response (i.e. negative PPI value). However, the correlations between PPI and GM volume in all three regions remained significant after this participant was excluded. The results therefore are presented for the entire sample. No other region across the entire brain met our criteria for a significant association with PPI with or without the latter participant.

The multiple regression analysis revealed that the (combined) structural volumes explained about 44% of the variance in PPI (*F* = 11.50, d.f. = 3,40, *p* < .001; *R*^2^ = .482; adjusted *R*^2^ = .440). The percentage of GM volumes at the maxima voxel in the three regions that were associated with PPI did not correlate with current age, age at illness onset, positive symptoms, negative symptoms or general psychopathology scores on the PANSS (all *p* > .05 uncorrected for multiple comparisons).

The relationships between the percentage of GM volumes at the maxima voxel in the three regions and 120-msec PPI (see Fig. 1) remained significant after we partialled out the effect of symptoms and mean pulse-alone amplitude [*r* (*p*) values for correlations between the left dorsolateral prefrontal, right middle frontal and orbital/medial frontal regions, respectively, and PPI: .498 (.001), .498 (.001), .469 (.002); after partialling out symptoms: .471 (.001), .501 (.001), .447 (.004); after partialling out overall pulse-alone amplitude: .406 (.009), .476 (.002), .360 (.021)]. The percentage of GM volume in the orbital/medial frontal cortex was correlated significantly positively with 30-msec (*r* = .302, *p* = .05) and 60-msec PPI (*r* = .457, *p* = .002); the associations between GM volumes in the left dorsolateral prefrontal and right middle frontal regions and 30-msec and 60-msec PPI were also in the same direction (positive) but not at a significant level (*r* values: .150–.232). Finally, the GM volume–PPI (120 msec) correlations after excluding patients under drugs other than atypical alone were very similar (*r* values for correlations between the left dorsolateral prefrontal, right middle frontal and right orbital/medial frontal regions, respectively, and PPI: .500, .507, .416) to that observed for the whole sample (presented earlier).

There was no association between total GM volume and 30-msec, 60-msec or 120-msec PPI (*r* values < .07).

## 4 Discussion

The investigation demonstrated an association between PPI and GM volume in keeping with the hypothesis with respect to the dorsolateral prefrontal (left), middle frontal (right), and the orbital/medial prefrontal (mainly right) cortices in patients with schizophrenia. In contrast, expected relationships between PPI and GM volume in other PPI-relevant regions, namely the hippocampus, temporal lobe, striatum, and thalamus, were not found.

The significant association found between the GM volume in the frontal cortex and PPI in patients with schizophrenia is not surprising since this region is part of the circuitry known to modulate PPI in experimental animals (reviews, Swerdlow and Geyer, 1998; Swerdlow et al., 2001) and has shown activation in association with PPI in functional neuroimaging studies of human subjects (Hazlett et al., 1998). Whilst PPI is considered primarily to be a pre-attentive mechanism (Graham, 1975), it may be susceptible to cognitive processes controlled in a ‘top down’ manner by the cortex (Hazlett and Buchsbaum, 2001). Of particular interest in this context is the observation that actively ‘attended’ prepulses produce more PPI than the ‘ignored’ ones, especially at prepulse-to-pulse intervals greater than 60 msec (Jennings et al., 1996; Dawson et al., 1997; Filion et al., 1998; Schell et al., 2000; Filion and Poje, 2003). However, active attention to prepulses is not necessary for PPI to occur (Blumenthal, 1999). Recent neuropsychological studies provide further evidence for an association between the prefrontal lobe and PPI in demonstrating greater PPI in healthy participants who also show superior performance on tasks that rely on the integrity and efficiency of prefrontal cortical function (Bitsios and Giakoumaki, 2005; Bitsios et al., 2006; Giakoumaki et al., 2006). More specifically, behavioural measures of planning, strategy formation, and selective attention are reported to covary with PPI in healthy people (Bitsios and Giakoumaki, 2005; Bitsios et al., 2006; Giakoumaki et al., 2006). Within the schizophrenia population, PPI shows small-to-modest positive correlation with performance on the Wisconsin Card Sort Test, which is widely regarded as a measure of planning and strategy formation (Butler et al., 1991; Kumari et al., 2007), and negative correlation with distractibility on the Continuous Performance Test (Karper et al., 1996; Kumari et al., 2007). Swerdlow et al. (2006) recently reported a positive association between PPI and functional status in patients with schizophrenia, which in turn may be a function of prefrontal cortex integrity (Wood et al., 2006), though a direct association between neuropsychological performance and PPI was not detected by these researchers. Our results showing associations between localised frontal lobe GM volumes and PPI, but no association between total GM volume and PPI, suggest specific and localised frontal cortex–PPI associations in people with schizophrenia.

Of the localised frontal regions found to show an association with PPI in the present study, the dorsolateral prefrontal cortex is considered vital for planning and strategy formation, and also implicated in selective attention (e.g. Banich et al., 2000a, 2000b; MacDonald et al., 2000; Peterson et al., 2002; Weiss et al., 2007). Both the dorsolateral as well as ventrolateral regions of the prefrontal cortex are strongly implicated in monitoring interference (Blasi et al., 2006). The right middle frontal gyrus is considered particularly involved in resolution of response competition (Hazeltine et al., 2003) and keeping ‘irrelevant information out of mind’ (Bunge et al., 2001). This latter finding is very pertinent to our observations since impaired inhibitory processes underlying diminished PPI in schizophrenia are conceptualized to reflect an overload of sensory information (Geyer et al., 1990; Braff, 1993).

The absence of a significant association between PPI and GM volume in the hippocampal, temporal, thalamic, and caudate regions (even when examined at a lower threshold) differs from our previous findings in healthy participants where GM volume in these regions was positively associated with PPI (Kumari et al., 2005). This difference is not likely to be due to a lack of power in the current study since it included a greater number of participants (*n* = 42) than our previous study (*n* = 24). Instead, other factors may be relevant. First, it has been suggested that behavioural abnormalities associated primarily with the temporal lobe are more strongly manifested in the presence of frontal lobe deficits in schizophrenia (Kurachi, 2003a, 2003b) and this, if true, may lead to stronger associations between such abnormalities and properties of the frontal lobe. Second, although prefrontal cortex hypofunctioning has been theoretically as well as empirically implicated in schizophrenia (Liddle et al., 1992; Andreasen et al., 1997; reviews, Kurachi, 2003a, 2003b), there is only modest evidence of prefrontal volumetric reduction (observed in 59% of studies reviewed; Shenton et al., 2001) with comparatively more robust evidence of structural alterations of medial temporal lobe structures (74% of studies reviewed), which include the amygdala, hippocampus, and parahippocampal gyrus, and neocortical temporal lobe regions (superior temporal gyrus; 100% of studies reviewed). It is plausible that there was relatively greater variance in the prefrontal lobe GM volume in our sample and this variance allowed a relationship with PPI to be detected whereas GM volume in the temporal lobe regions may have been relatively more compromised across the entire sample. The evidence for thalamus volume reduction is also moderate (review, Shenton et al., 2001), but this brain region may relate more strongly to normal and deficient PPI at the functional than at the structural level as discussed previously (Kumari et al., 2005). Third, and perhaps the most likely, reason may be that behavioural parameters normally associated with volumes of the temporal lobe, hippocampus and parahippocampal gyrus remain associated with volumes of these regions in female patients while such associations may be disrupted in male patients with schizophrenia (review, Antonova et al., 2004). Conversely, prefrontal cortex volume and behavioural measures of its function do not appear to be influenced by gender in patients with schizophrenia (review, Antonova et al., 2004). The fact that the present study included male patients only may be one reason why we observed a significant association of PPI to GM volume in the frontal cortex but not in some other PPI-relevant regions.

In total, GM volume in the frontal lobe explained about 44% of variance in PPI in patients in this study whereas about 62% of the variance in PPI was explained by GM volume in multiple cortical and limbic regions in our previous study of healthy participants (Kumari et al., 2005). There may be a stronger structure–function correspondence in healthy people than in people (especially males) with schizophrenia (Antonova et al., 2004). Furthermore, several other factors, such as the presence of thought disorder (Perry and Braff, 1994; Perry et al., 1999; Meincke et al., 2004) and brain abnormalities at the functional level (Hazlett et al., 1998; Kumari et al., 2003, 2007) are also known to be associated with deficient PPI in schizophrenia. The strength of GM volume–PPI correlations when examined in patients treated with atypical antipsychotics alone was very similar to that observed for the entire sample. This may reflect the fact that the use of atypical antipsychotics is associated with both increased cortical GM volume (Garver et al., 2005; Lieberman et al., 2005) and increased PPI in people with schizophrenia (reviews, Kumari and Sharma, 2002; Swerdlow et al., 2006; Kumari et al., 2007).

In conclusion, our findings indicate that sensorimotor gating function in schizophrenia is facilitated by availability of neural resources in the frontal cortex. Future studies are needed to examine PPI–GM volume relationships in both male and female patients with schizophrenia.

## Figures and Tables

**Table 1:** Participant characteristics (*n* = 42)

	Mean (SD)
Age (in years)	37.9 (9.50)

Premorbid (NART) IQ	107.84 (8.97)
Current IQ	101.76 (9.04)
Education (in years)	13.50 (2.30)

Age at illness onset (in years)^a^	24.22 (7.51)
Duration of illness (years)	14.32 (10.51)

*Symptoms*	
PANSS: positive	17.34 (4.44)
PANSS: negative	18.37 (4.26)
PANSS: general psychopathology	33.61 (6.52)

Total	69.32 (12.97)

*Diagnosis (n)*	
Paranoid schizophrenia	32
Residual schizophrenia	3
Schizophrenia undifferentiated	1
Schizoaffective disorder	6

*Current antipsychotic medication type (n)*	
Atypical	34
Typical	6
Both	2

	Mean (SEM)

*PPI (% inhibition)*	
30 msec	11.26 (4.59)
60 msec	22.62 (4.13)
120 msec	32.26 (4.94)

*Startle amplitude (in arbitrary units)*	202.35 (25.74)

*Latency to peak (in msec)*	
Pulse-alone	69.32 (1.24)
30 msec	65.13 (1.59)
60 msec	65.01 (2.09)
120 msec	65.07 (2.12)

NART: National Adult Reading Test (Nelson and Willison, 1991). PANSS: Positive and Negative Syndrome Scale (Kay et al., 1987).

a *n* = 41, Reliable information about the age at illness onset was unavailable for one participant.

## References

[bib1] Andreasen N.C., O'leary D.S., Flaum M., Nopoulos P., Watkins G.L., Boles Ponto L.L., Hichwa R.D. (1997). Hypofrontality in schizophrenia: distributed dysfunctional circuits in neuroleptic-naive patients. Lancet.

[bib2] Antonova E., Sharma T., Morris R., Kumari V. (2004). The relationship between brain structure and neurocognition in schizophrenia: a selective review. Schizophrenia Research.

[bib3] Ashburner J., Friston K.J. (1999). Nonlinear spatial normalization using basis functions. Human Brain Mapping.

[bib4] Ashburner J., Friston K.J. (2000). Voxel-based morphometry – the methods. NeuroImage.

[bib5] Banich M.T., Milham M.P., Atchley R.A., Cohen N.J., Webb A., Wszalek T., Kramer A.F., Liang Z., Barad V., Gullett D., Shah C., Brown C. (2000). Prefrontal regions play a predominant role in imposing an attentional ‘set’: evidence from fMRI. Cognitive Brain Research.

[bib6] Banich M.T., Milham M.P., Atchley R., Cohen N.J., Webb A., Wszalek T., Kramer A.F., Liang Z.P., Wright A., Shenker J., Magin R. (2000). fMRI studies of Stroop tasks reveal unique roles of anterior and posterior brain systems in attentional selection. Journal of Cognitive Neuroscience.

[bib7] Blumenthal T.D., Dawson M.E., Schell A.M., Böhmelt A.H. (1999). Short lead interval startle modification. Startle Modification: Implications for Neuroscience, Cognitive Science and Clinical Science.

[bib8] Bitsios P., Giakoumaki S.G. (2005). Relationship of prepulse inhibition of the startle reflex to attentional and executive mechanisms in man. International Journal of Psychophysiology.

[bib9] Bitsios P., Giakoumaki S.G., Theou K., Frangou S. (2006). Increased prepulse inhibition of the acoustic startle response is associated with better strategy formation and execution times in healthy males. Neuropsychologia.

[bib10] Blasi G., Goldberg T.E., Weickert T., Das S., Kohn P., Zoltick B., Bertolino A., Callicott J.H., Weinberger D.R., Mattay V.S. (2006). Brain regions underlying response inhibition and interference monitoring and suppression. European Journal of Neuroscience.

[bib11] Braff D.L. (1993). Information processing and attention dysfunctions in schizophrenia. Schizophrenia Bulletin.

[bib12] Braff D.L., Geyer M.A., Swerdlow N.R. (2001). Human studies of prepulse inhibition of startle: normal subjects, patient groups, and pharmacological studies. Psychopharmacology.

[bib13] Braff D.L., Light G.A., Ellwanger J., Sprock J., Swerdlow N.R. (2005). Female schizophrenia patients have prepulse inhibition deficits. Biological Psychiatry.

[bib14] Braff D.L., Stone C., Callaway E., Geyer M., Glick I., Bali L. (1978). Prestimulus effects on human startle reflex in normals and schizophrenics. Psychophysiology.

[bib15] Bunge S.A., Ochsner K.N., Desmond J.E., Glover G.H., Gabrieli J.D. (2001). Prefrontal regions involved in keeping information in and out of mind. Brain.

[bib16] Butler R.W., Jenkins M.A., Geyer M.A., Braff D.L., Tamminga C.A., Schulz S.C. (1991). Wisconsin card sorting deficits and diminished sensorimotor gating in a discrete subgroup of schizophrenic patients.

[bib17] Dawson M.E., Schell A.M., Swerdlow N.E., Filion D.L., Lang P.J., Simons R.F., Balaban M. (1997). Cognitive, clinical, and neurophysiological implications of startle modification. Attention and Orienting: Sensory and Motivational Processes.

[bib18] Ettinger U., Kumari V., Chitnis X.A., Corr P.J., Sumich A.L., Rabe-Hesketh S., Crawford T.J., Sharma T. (2002). Relationship between brain structure and saccadic eye movements in healthy humans. Neuroscience Letters.

[bib19] Ettinger U., Kumari V., Chitnis X.A., Corr P.J., Crawford T.J., Fannon D.G., O'ceallaigh S., Sumich A.L., Doku V.C., Sharma T. (2004). Volumetric neural correlates of antisaccade eye movements in first-episode psychosis. American Journal of Psychiatry.

[bib20] Ettinger U., Antonova E., Crawford T.J., Mitterschiffthaler M., Sharma T., Kumari V. (2005). Structural neural correlates of prosaccade and antisaccade eye movements in healthy humans. NeuroImage.

[bib21] Filion D.L., Dawson M.E., Schell A.M. (1998). The psychological significance of human startle eyeblink modification: a review. Biological Psychology.

[bib22] Filion D.L., Poje A.B. (2003). Selective and nonselective attention effects on prepulse inhibition of startle: a comparison of task and no-task protocols. Biological Psychology.

[bib23] First M.B., Spitzer R.L., Gibbon M., Williams J.B.W. (1995). Structured Clinical Interview for DSM-IV Axis I Disorders, Patient Edition (SCID-P), Version 2.

[bib24] Gaser C., Schlaug G. (2003). Brain structures differ between musicians and non-musicians. Journal of Neuroscience.

[bib25] Garver D.L., Holcomb J.A., Christensen J.D. (2005). Cerebral cortical gray expansion associated with two second generation antipsychotics. Biological Psychiatry.

[bib26] Geyer M.A., Swerdlow N.R., Mansbach R.S., Braff D.L. (1990). Startle response models of sensorimotor gating and habituation deficits in schizophrenia. Brain Research Bulletin.

[bib27] Geyer M.A., Krebs-Thomson K., Braff D.L., Swerdlow N.R. (2001). Pharmacological studies of prepulse inhibition models of sensorimotor gating deficits in schizophrenia: a decade in review. Psychopharmacology.

[bib28] Giakoumaki S.G., Bitsios P., Frangou S. (2006). The level of prepulse inhibition in healthy individuals may index cortical modulation of early information processing. Brain Research.

[bib29] Good C.D., Johnsrude I.S., Ashburner J., Henson R.N., Friston K.J., Frackowiak R.S. (2001). A voxel-based morphometric study of ageing in 465 normal adult human brains. NeuroImage.

[bib30] Graham F.K. (1975). The more or less startling effects of weak prestimuli. Psychophysiology.

[bib31] Hazeltine E., Bunge S.A., Scanlon M.D., Gabrieli J.D. (2003). Material-dependent and material-independent selection processes in the frontal and parietal lobes: an event-related fMRI investigation of response competition. Neuropsychologia.

[bib32] Hazlett E.A., Buchsbaum M.S. (2001). Sensorimotor gating deficits and hypofrontality in schizophrenia. Frontiers in Bioscience.

[bib33] Hazlett E.A., Buchsbaum M.S., Haznedar M.M., Singer M.B., Germans M.K., Schnur D.B., Jimenez E.A., Buchsbaum B.R., Troyer B.T. (1998). Prefrontal cortex glucose metabolism and startle eyeblink modification abnormalities in unmedicated schizophrenia patients. Psychophysiology.

[bib34] Jennings P.D., Schell A.M., Filion D.L., Dawson M.E. (1996). Tracking early and late stages of information processing: contributions of startle eyeblink reflex modification. Psychophysiology.

[bib35] Karper L.P., Freeman G.K., Grillon C., Morgan CA Charney D.S., Krystal J.H. (1996). Preliminary evidence of an association between sensorimotor gating and distractibility in psychosis. Journal of Neuropsychiatry and Clinical Neurosciences.

[bib36] Kay S.R., Fishbein A., Olper L.A. (1987). The positive and negative syndrome scale. Schizophrenia Bulletin.

[bib37] Kumari V., Antonova E., Geyer M.A., Ffytche D., Williams S.C., Sharma T. (2007). A fMRI investigation of startle gating deficits in schizophrenia patients treated with typical or atypical antipsychotics. International Journal of Neuropsychopharmacology.

[bib38] Kumari V., Antonova E., Zachariah E., Galea A., Aasen I., Ettinger U., Mitterschiffthaler M.T., Sharma T. (2005). Structural brain correlates of prepulse inhibition of the acoustic startle response in healthy humans. NeuroImage.

[bib39] Kumari V., Gray J.A., Geyer M.A., Ffytche D., Mitterschiffthaler M.T., Vythelingum G.N., Williams S.C.R., Simmons A., Sharma T. (2003). Neural correlates of prepulse inhibition in normal and schizophrenic subjects: a functional MRI Study. Psychiatry Research: Neuroimaging.

[bib40] Kumari V., Ettinger U., Lang M.V. (2005). Prepulse inhibition deficits in schizophrenia: static or amenable to treatment?. Progress in Schizophrenia Research.

[bib41] Kumari V., Sharma T. (2002). Effects of typical and typical antipsychotics on prepulse inhibition in schizophrenia: a critical evaluation of current evidence and directions for future research. Psychopharmacology.

[bib42] Kumari V., Soni W., Sharma T. (2001). Influence of cigarette smoking on prepulse inhibition of the acoustic startle response in schizophrenia. Human Psychopharmacology.

[bib43] Kurachi M. (2003). Pathogenesis of schizophrenia: part I. Symptomatology, cognitive characteristics and brain morphology. Psychiatry and Clinical Neurosciences.

[bib44] Kurachi M. (2003). Pathogenesis of schizophrenia: part II. Temporo-frontal two-step hypothesis. Psychiatry and Clinical Neurosciences.

[bib45] Liddle P.F., Friston K.J., Frith C.D., Hirsch S.R., Jones T., Frackowiak R.S. (1992). Patterns of cerebral blood flow in schizophrenia. British Journal of Psychiatry.

[bib46] Lieberman J.A., Tollefson G.D., Charles C., Zipursky R., Sharma T., Kahn R.S., Keefe R.S., Green A.I., Gur R.E., McEvoy J., Perkins D., Hamer R.M., Gu H., Tohen M., HGDH Study Group (2005). Antipsychotic drug effects on brain morphology in first-episode psychosis. Archives of General Psychiatry.

[bib47] Maguire E.A., Gadian D.G., Johnsrude I.S., Good C.D., Ashburner J., Frackowiak R.S., Frith C.D. (2000). Navigation-related structural change in the hippocampi of taxi drivers. Proceedings of the National Academy of Sciences of the United States of America.

[bib48] MacDonald A.W., Cohen J.D., Stenger V.A., Carter C.S. (2000). Dissociating the role of the dorsolateral prefrontal and anterior cingulate cortex in cognitive control. Science.

[bib49] Meincke U., Mörth D., Voß T., Thelen B., Geyer M.A., Gouzoulis-Mayfrank E. (2004). Prepulse inhibition of the acoustically evoked startle reflex in patients with an acute schizophrenic psychosis – a longitudinal study. European Archives of Psychiatry and Clinical Neuroscience.

[bib50] Nelson H.E., Willison J. (1991). National Adult Reading Test (NART) Test Manual.

[bib51] Perry W., Geyer M.A., Braff D.L. (1999). Sensorimotor gating and thought disturbance measured in close temporal proximity in schizophrenic patients. Archives of General Psychiatry.

[bib52] Perry W., Braff D.L. (1994). Information-processing deficits and thought disorder in schizophrenia. American Journal of Psychiatry.

[bib53] Peterson B.S., Kane M.J., Alexander G.M., Lacadie C., Skudlarski P., Leung H.C., May J., Gore J.C. (2002). An event-related functional MRI study comparing interference effects in the Simon and Stroop tasks. Cognitive Brain Research.

[bib54] Sanfilipo M., Lafargue T., Rusinek H., Arena L., Loneragan C., Lautin A., Rotrosen J., Wolkin A. (2002). Cognitive performance in schizophrenia: relationship to regional brain volumes and psychiatric symptoms. Psychiatry Research.

[bib55] Schell A.M., Wynn J.K., Dawson M.E., Sinaii N., Niebala C.B. (2000). Automatic and controlled attentional processes in startle eyeblink modification: effects of habituation of the prepulse. Psychophysiology.

[bib56] Shenton M.E., Dickey C.C., Frumin M., McCarley R.W. (2001). A review of MRI findings in schizophrenia. Schizophrenia Research.

[bib57] Sowell E.R., Thompson P.M., Holmes C.J., Batth R., Jernigan T.L., Toga A.W. (1999). Localizing age-related changes in brain structure between childhood and adolescence using statistical parametric mapping. NeuroImage.

[bib58] Swerdlow N.R., Light G.A., Cadenhead K.S., Sprock J., Hsieh M.H., Braff D.L. (2006). Startle gating deficits in a large cohort of patients with schizophrenia: relationship to medications, symptoms, neurocognition, and level of function. Archives of General Psychiatry.

[bib59] Swerdlow N.R., Geyer M.A. (1998). Using an animal model of deficient sensorimotor gating to study the pathophysiology and new treatments of schizophrenia. Schizophrenia Bulletin.

[bib60] Swerdlow N.R., Geyer M.A., Braff D.L. (2001). Neural circuit regulation of prepulse inhibition of startle in the rat: current knowledge and future challenges. Psychopharmacology.

[bib61] Wechsler D. (1999). Wechsler Abbreviated Scale of Intelligence.

[bib62] Weiss E.M., Siedentopf C., Golaszewski S., Mottaghy F.M., Hofer A., Kremser C., Felber S., Fleischhacker W.W. (2007). Brain activation patterns during a selective attention test – a functional MRI study in healthy volunteers and unmedicated patients during an acute episode of schizophrenia. Psychiatry Research.

[bib63] Wood S.J., Berger G.E., Lambert M., Conus P., Velakoulis D., Stuart G.W., Desmond P., McGorry P.D., Pantelis C. (2006). Prediction of functional outcome 18 months after a first psychotic episode: a proton magnetic resonance spectroscopy study. Archives of General Psychiatry.

